# PPARs as Metabolic Sensors and Therapeutic Targets in Liver Diseases

**DOI:** 10.3390/ijms22158298

**Published:** 2021-08-02

**Authors:** Hugo Christian Monroy-Ramirez, Marina Galicia-Moreno, Ana Sandoval-Rodriguez, Alejandra Meza-Rios, Arturo Santos, Juan Armendariz-Borunda

**Affiliations:** 1 Instituto de Biologia Molecular en Medicina, Centro Universitario de Ciencias de la Salud, Universidad de Guadalajara, Guadalajara 44340, Jalisco, Mexico; hugo.monroyram@academicos.udg.mx (H.C.M.-R.); marina.galicia@academicos.udg.mx (M.G.-M.); anasol44@hotmail.com (A.S.-R.); 2 Tecnologico de Monterrey, Escuela de Medicina y Ciencias de la Salud, Zapopan 45138, Jalisco, Mexico; alejandramezarios@yahoo.com.mx (A.M.-R.); arturo.santos@tec.mx (A.S.)

**Keywords:** metabolic alterations, hepatic damage, nuclear factors, pharmacological targets

## Abstract

Carbohydrates and lipids are two components of the diet that provide the necessary energy to carry out various physiological processes to help maintain homeostasis in the body. However, when the metabolism of both biomolecules is altered, development of various liver diseases takes place; such as metabolic-associated fatty liver diseases (MAFLD), hepatitis B and C virus infections, alcoholic liver disease (ALD), and in more severe cases, hepatocelular carcinoma (HCC). On the other hand, PPARs are a family of ligand-dependent transcription factors with an important role in the regulation of metabolic processes to hepatic level as well as in other organs. After interaction with specific ligands, PPARs are translocated to the nucleus, undergoing structural changes to regulate gene transcription involved in lipid metabolism, adipogenesis, inflammation and metabolic homeostasis. This review aims to provide updated data about PPARs’ critical role in liver metabolic regulation, and their involvement triggering the genesis of several liver diseases. Information is provided about their molecular characteristics, cell signal pathways, and the main pharmacological therapies that modulate their function, currently engaged in the clinic scenario, or in pharmacological development.

## 1. Introduction

The liver is the main organ responsible for biochemical metabolism in the human body, compounds absorbed by the intestine such as nutrients or drugs, first pass through the liver, where they are processed into simpler products, maintaining and regulating their levels in the bloodstream [1]. Carbohydrates and lipids are two components of the diet that are metabolized by the liver to generate the necessary energy, leading to several physiological processes that help maintain body homeostasis [2]. However, a dysfunction in hepatic metabolism can result in the genesis of several hepatic diseases such as_MAFLD, ALD, fibrosis/cirrhosis, viral hepatitis by hepatitis B (HBV) or hepatitis C(HCV) infection, or in some cases HCC [2,3].

Peroxisome proliferator-activated receptors (PPARs) are a family of ligand-dependent transcription factors that regulate essential metabolic processes in the liver and other organs where they are activated by endogenous ligands such as fatty acids and similar compounds. Three isoforms of PPARs are known: PPARα, PPARβ/δ, and PPARγ, all of them with different distribution, affinity and specificity for their agonists, and the ability to modulate lipid metabolism and energy homeostasis in mammals [4]. All the changes that occur during liver injury alter metabolic functionality, aggravate liver damage, and make PPARs important therapeutic targets for the treatment of these diseases [5].

## 2. Overviews of PPARs α, β/δ and γ

As we previously mentioned, PPARs are transcription factors of nuclear hormone receptor, a family composed by three subtypes: PPARα, PPARβ/δ, and PPARγ; each encoded by a different gene located in different chromosomes and characterized by different distribution patterns and specific ligands [6,7,8]. In this section, we describe the molecular characteristics and functions of each subtype.

### 2.1. Structure and Molecular Characteristics

**Figure 1:**
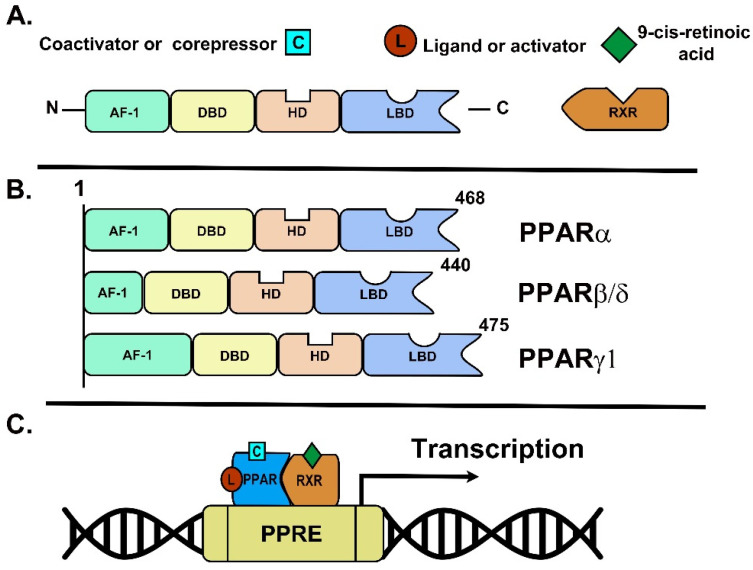
Representation of PPARs’ structure and their molecular function. (**A**) The universal structure of PPARs is represented by AF-1, DBD, HD and LBD domains. The active form of PPARs is heterodimerizing with RXR in conjunction with ligands, co-activators and co-repressors that modulate their function. (**B**) PPARs’ subtypes representations. (**C**) Transcription of PPARs target genes start upon the union of PPAR-RXR and the ligands and co-activators into PPRE sequences.

Structurally, PPARs are similar to steroid and thyroid hormone receptors, and they can be stimulated by small lipophilic ligands [9]. In general, the three-dimensional structure of PPARs consists of a canonical domain that is shared with other nuclear receptors, including the amino-terminal AF-1 trans activation domain (A/B domain); a DNA-binding domain (DBD or C domain) in their N-terminus containing two highly conserved zinc finger motifs with globular structure; and a dimerization and ligand-binding domain (LBD or E/F domain) with a ligand-dependent transactivation function AF-2 (promotes the recruitment of co-activators) at the carboxy-terminal region, which is responsible for ligand specificity and PPAR activation binding to the peroxisome proliferator response elements (PPRE) [7,8,9,10,11] (Figure 1A). The LBD is characterized by its size, which is larger than other nuclear receptors; this feature allows a wide range of unsaturated fatty acids to bind [7,10,12]. In addition, PPARs contain a hinge region functioning as a docking site for cofactors (D domain) [8,11]. PPARs’ subtype structures are illustrated in Figure 1B.

### 2.2. Mechanisms of Action

After interaction with the specific ligands, PPARs are translocated to the nucleus, and heterodimerizes with another nuclear receptor; the retinoid X receptor (RXR), which binds to PPAR through two zinc fingers in the DBD, specifically PPREs present in the vicinity of PPAR-responsive genes promoters, subsequently altering co-activator/co-repressor dynamics to modulate transcription [7,8,10]. PPREs generally have a direct repeat of hexanucleotide core recognition elements (5′AGGTCA-3′) spaced by one or two bp [11]. In addition, the hexanucleotide core has an extension 5′-AACT that provides polarity for heterodimer binding with RXR [12]. Once activated, the heterodimer PPARs/RXR recruit different nuclear receptor co-factors and gene transcription initiates [11] (Figure 1C). In addition, PPAR co-activators are cAMP response element-binding protein, steroid receptor coactivator-1, and the PPARγ coactivator 1α. In addition, co-repressors comprise the nuclear receptor co-repressor, and silence the mediator of the retinoid and thyroid hormone receptor [8,11].

The activation of PPARα and PPARβ/δ mostly facilitates energy combustion and the activation of PPARγ contributes to energy storage [10]. Table 1 summaries the main characteristics of each PPAR subtype.

#### Signal Pathways

**Figure 2:**
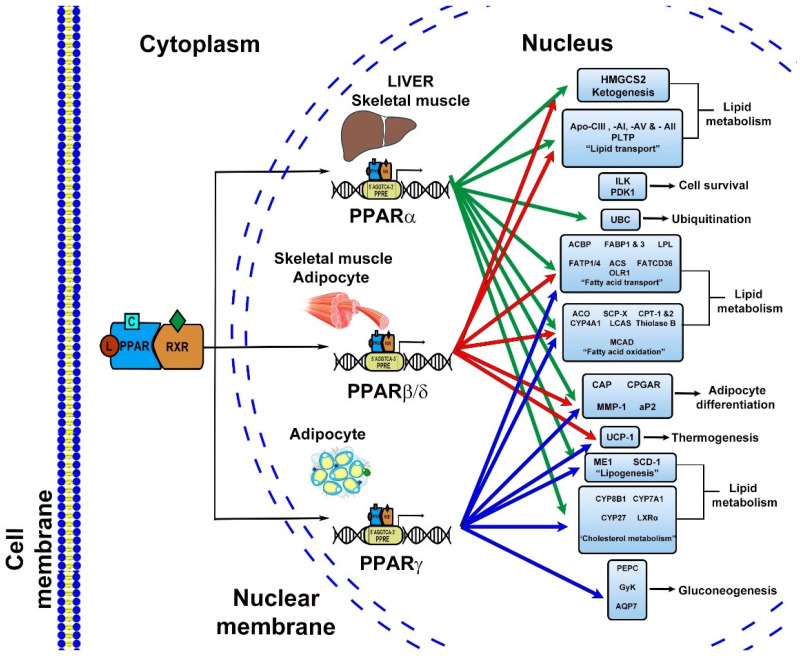
PPARs’ signal transduction. The signal pathways where PPARs are involved affects gluconeogenesis, lipid metabolism, thermogenesis, adipocyte differentiation, ubiquitination and cell survival, depending on the target gene and tissues where they are activated.

PPARs activated different signal pathways, mainly via endogenous ligands products of the metabolic pathways of fatty acids, which is the reason why they are called lipid sensors [10]. The main signal transduction pathways related with different PPARs subtypes are recapitulated in Figure 2.

## 3. Role of PPARs in Liver Diseases

The most shocking liver diseases worldwide are MAFLD, fibrosis, HCC, HBV and (HCV infections, along with ALD. Several of them, such as HCC, represent a major cause of mortality in the world. Because of this, it is necessary to understand PPARs role as metabolic regulators in the development of these pathologies, and design new effective ligands able to modulate the activity of these receptors, minimizing side effects. The main functions of PPARs in each one of the aforesaid diseases will be described below

### 3.1. Gene Expression Alterationof PPARs in MAFLD

MAFLD, formerly named non-alcoholic fatty liver disease (NAFLD), affects 20–30% of adult population in western countries [21]. This damage is characterized by hepatic steatosis accompanied by one of three features: overweight or obesity, T2DM, or lean or normal weight with evidence of metabolic dysregulation [22]. During MAFLD, elevated level of circulating free fatty acids increases fat influx into hepatocytes, causing an augmented fatty acid oxidation in mitochondria and peroxisomes leading to ROS generation, causing oxidative stress [23]. Imbalance between ROS generation and antioxidant mechanisms leads to mitochondrial and peroxisome dysfunction; and eventually to apoptosis of hepatocytes exacerbating the proinflammatory events of non-alcoholic steatohepatitis (NASH). Peroxisomes and mitochondria jointly perform various metabolic roles including O_2_ and lipid metabolism; these organelles are indispensable in a healthy liver for the breakdown of long-chain fatty acids, very-long-chain fatty acids, and branched-chain fatty acids through α and β-oxidation. Subsequently, they prevent the accumulation of fatty acids (FAs) in the liver. In addition, Acyl-coenzyme A oxidase enzyme (ACOX1) is a rate-limiting enzyme of peroxisomal beta-oxidation of long chain fatty acids exclusive of peroxisomes, alterations in ACOX1 results in hepatic steatosis [24].

#### 3.1.1. PPARα

Of all PPARs; PPARα is the most relevant one to NASH pathogenesis; since it is a metabolic sensor upregulated by fasting and responsible for transcriptional upregulation of β-oxidation genes [25], then altered expression of this transcription factor induces lipogenesis. Therefore, PPARα agonists are potential targets for NASH treatment. Peroxisome biogenesis and proliferation are also regulated by PPARα. After the activation this nuclear transcription factor, the expression of several genes encoding for peroxisomal proteins and genes controlling beta oxidation, fatty acid uptake, triglyceride turnover, bile acid synthesis, adipogenesis, ketogenesis, glucose metabolism and adipocyte differentiation are induced [10,26,27,28]. Additionally, PPARα exerts anti-inflammatory effects through a negative crosstalk with NF-κB and AP-1 (Activator Protein 1) [29]. In normal conditions, hepatocytes have a high expression of PPARα. In NASH patients, hepatic expression of PPARα decreases and negatively correlates with the severity of the disease [30]. Correspondingly, several authors have reported that expression of PPARα is reduced in NASH models [31,32]. PPARα reduction is believed to be due to an increased expression/activation of Rho-associated protein kinase (ROCK) and to a reduction in peroxisomes number caused by elevated ROS in NASH [33,34]. Hepatic decrease in PPARα expression causes a deficiency in the transcription of its target gene carnitine palmitoyl transferase 1 (CPT-1A) and excessive FAs tend to accumulate in the form of triglycerides, since they cannot go through the inner mitochondrial membrane and reach the mitochondrial matrix for further metabolism [35].

Levels of PPARα are recovered in NASH with statins [31] due to a reduced RhoA cell membrane translocation. Additionally, PPARα increases as a result of lifestyle change or bariatric surgery with the improvement of histological NAFLD score [30].

Intriguingly, some PPARα properties, such as increased DNA synthesis and peroxisome pro-liberation, are observed only in mice and rats, but not in humans. This could be due to the ten-fold higher expression of PPARα in the liver of rodents, that can also partially explain differences in the efficacy of PPARα agonists in experimental models against human studies. Interestingly, it has been suggested that increased hepatic expression of PPARα and target genes involved in fatty acid oxidation is a protective response to high fat diet [36,37,38].

Mice deficient in PPARα are susceptible to dietary fat-mediated NASH, oxidative stress, cell death and hepatic inflammation [36,39,40,41]. Pharmacological activation of PPARα in the methionine–choline-deficient diet (MCDD) NASH model, reduces lipid peroxidation and TG content in the liver, and reverses steatohepatitis and fibrosis [42,43]. Additionally, PPARα agonists prevent dietary steatohepatitis by a direct effect on inflammation, independent of its effect on lipid accumulation in hepatocytes and independent of PPARα binding to PPREs [44]. Additionally, Pirfenidone seem to be a PPARα agonist that improves NASH through SIRT1/LKB1/pAMPK signaling [32].

#### 3.1.2. PPARβ/δ

PPARβ/δ is well expressed in hepatocytes, Kupffer cells (KCs) and hepatic stellate cells (HSCs). Most of the work to study MAFLD has been conducted with PPARβ/δ agonist. In MCDD-fed mice, the treatment with PPARδ agonist GW501516 increased hepatic expression of ACOX1, CPT-1A and FABP1 (liver fatty acid binding protein); and decreased hepatic triglyceride content [45]. In obese monkeys, GW501516 normalized serum insulin and TAG concentrations, decreased low-density/lipoprotein cholesterol, and increased high-density lipoprotein cholesterol [46]. Additionally, PPARβ/δ agonist has been shown in high fat diet-fed mice to favor a slender phenotype, improved insulin sensitivity, and prevent hepatic lipid accumulation, due to higher rates of energy expenditure [47]. It also favors an upregulation of Adfp and Cpt1a and enhanced FA oxidation, as well as activation of AMPK and inhibition of sterol regulatory element-binding protein 1c (SREBP-1c), reducing hepatic lipogenesis using GW501516 [48].

In 2008, Riserus et al. published a paper confirming that GW501516 reduced liver fat content, and TG, LDL, ApoB and insulin in plasma in moderate obese men and muscle expression of CPT1b was also significantly increased [49]. Other PPARβ/δ agonists tested in overweight subjects with dyslipidemia demonstrated diminution in GGT and favorable lipid profiles [50]. Adenovirus mediated upregulation of PPARβ/δ into db/db mice resulted in the activation of SREBP-1c, upregulation of lipase, and improved liver steatosis [51]. Similarly, Liu et al. found increased hepatic expression of ACC1, FA uptake and beta oxidation [52]. Quite the opposite, PPARδ-null mice had lower metabolic activity and glucose intolerance [53]. Use of hepatocyte-specific PPARδ null mice identified that hepatic PPARβ/δ augments FA muscle utilization and improves dyslipidemia through a metabolic network between hepatic PPARβ/δ and muscle PPARα. Up to now, there is not enough evidence that PPARβ/δ clinical intervention can be effective for the treatment of MALFD, and carcinogenesis remain a concern.

#### 3.1.3. PPARγ

PPARγ is mostly known by its role in regulation of adipocyte differentiation and high expression of adipose tissue and macrophages. However, hepatic PPARγ expression is robustly induced in NAFLD patients and experimental models [54,55,56,57]. Increased activation of PPARγ downregulates the expression of pro-inflammatory cytokines, such as TNF-α, IFN-γ, IL-2, IL-1β, IL-6, MCP-1, and MIP-1β [57] and results in a decreased activation of TLR-4 pathway. In contrast, activation of TLR-4 pathway leads to the downregulation of PPARγ by negatively interfering with NF-kB in macrophages [58]. Additionally, it polarizes macrophages into anti-inflammatory M2 phenotype [59].

PPARγ upregulates proteins associated with lipid uptake, TAG storage, and the formation of lipid droplets, such as FABP4, fat-specific protein 27 (FSP27)/Cidec, CD36, monacylglycerol O-acyltransferase 1, and perilipin 2; then PPARγ hepatic expression promotes steatosis. Overexpression of PPARγ2 in hepatocytes increased steatosis; on the contrary, in mice hepatocyte-specific PPARγ-deficient (Pparγ-DHEP) hepatosteatosis was decreased [60,61,62]. In Pparγ-DHEP mice, liver expression of genes associated with adipogenesis, and FA uptake were downregulated, but systemic insulin resistance, adiposity, and hyperlipidemia were aggravated [56]. In HFD-fed animals, Pparγ and Srebp1c, CD36 and FAS are overexpressed. Even though activation of PPARγ is steatogenic, treatment with PPARγ ligands to genetically obese or diet-induced NASH mice decreases hepatic TAG due to adiponectic-mediated glucose uptake and AMPK activation, thereby improving FA oxidation in hepatocytes [62]. Thiazolidinediones (TZD), also called glitazones, are PPARγ-ligands and in the absence of adipose tissue, the liver is the primary target for TZD action. Clinical trials utilizing TZDs showed significant improvement in hepatic steatosis and inflammation. However, weight gain concerns and other side effects remain.

#### 3.1.4. Clinical Trials of PPAR-Related Drugs in NASH

Elafibranor is a well-known dual PPARα/δ agonist. A Phase-2b Golden-505 study has demonstrated that, in NASH patients without cirrhosis treated with 120 mg daily for 52 weeks, insulin, sensitivity, glucose homeostasis, and lipid metabolism were improved and inflammation reduced [63]. However, in phase III study, Genfit announced interim results after 72 weeks in RESOLVE-IT study which showed that the trial did not meet histological improvement or NASH resolution without worsening of fibrosis in the ITT intention to treat (ITT) population of 1070 patients with nonalcoholic steatohepatitis (NASH) and fibrosis (https://www.natap.org/2020/AASLD/AASLD_162.htm) (accessed on 30 July 2021).

FXR agonists are used to treat non-alcoholic fatty liver disease (NAFLD), in part because they reduce hepatic lipids.

Obeticholic acid (OCA) is a selective and potent agonists for the farnesoid X receptor (FXR). FXR-PPARγ cascade has demonstrated clinical efficacy in NASH. In the phase 3 double-blind, randomized, placebo-controlled, multicenter, REGENERATE study, it was demonstrated that after 18 months OCA significantly improved fibrosis in 1218 noncirrhotic NASH patients using 25 mg. Additionally, Nonalcoholic Fatty Liver Disease Activity Score decreased (by ≥2 points), and quality of life was impaired, or NASH resolution had greater patient-reported outcomes (PROs) improvements in some domains (ClinicalTrials.gov, Number NCT02548351, https://doi.org/10.1016/j.cgh.2021.07.020) (accessed on 30 July 2021).

The thiazolidinedione class of insulin-sensitizing drugs, including rosiglitazone and pioglitazone, are potent pharmacologic PPARγ agonists. Thiazolidinediones increase plasma adiponectin levels in DM2 and NASH patients [64,65] and levels became similar to the values observed in control subjects. Pioglitazone treatment increases adiponectin concentrations, and improves hepatic insulin resistance and liver histology in NASH [66]. In NASH patients treated with pioglitazone, a reduction was observed in hepatic steatosis but also necroinflammation and fibrosis [67]. It has been suggested that adiponectin may play an important role in mediating the beneficial effects of pioglitazone in NASH patients, inhibiting hepatic fatty acid synthesis, gluconeogenesis and de novo lipogenesis, via activation of AMPK. It also activates PPARα with the stimulation of fatty acid oxidation [68].

Ianifibranor (IVA337) is a next-generation pan-PPAR agonist addressing the pathophysiology of NASH: metabolic, inflammatory and fibrotic. A phase 2b study aiming to evaluate the efficacy and the safety of two doses of IVA337 (800 mg, 1200 mg) per day for 24 weeks versus placebo in adult NASH patients with liver steatosis and moderate to severe necroinflammation without cirrhosis demonstrated that SAF Activity Score (SAF-A) decrease at least 2 points (SAF histological score, calculated as the sum of lobular inflammation score and balloning score) with stable or decreases CRN Fibrosis Score (CRN-F) (Clinical Trial NCT03008070).

### 3.2. PPARs Expression in Liver Fibrosis

Hepatic fibrosis results from a chronic inflammatory process that affects hepatocytes or biliary cells. Inflammation leads to the activation of effector cells, which results in the accumulation of extracellular matrix components, such as collagens. In liver, HSCs appear to be the primary source of extracellular matrix. These cells change its normal function as a retinol storage cell to a proliferative, contractile and myofibroblastic-like phenotype [69]. Persistent fibrosis leads to cirrhosis—a pathology with an ominous parenchymal lesion and many clinical complications—and even to HCC. Numerous molecular pathways are involved in fibrosis development, but one mainly important is TGF-β pathway. TGF-β is a pleitrotropic cytokine involved as the dominant stimuli for HSCs to produce extracellular matrix (ECM) wound-components and is increased in experimental and clinical fibrosis and its expression is regulated mostly through Smads signaling [70]. A pathway that seems exclusive to liver fibrosis comprises Toll-like receptor 4 (TLR4). TLR4 is activated on HSCs surface by lipopolysaccharides derived from translocated intestinal bacteria, triggering cell activation and fibrogenesis.

#### 3.2.1. PPARα

PPARα is not expressed in rodent or human HSCs [71]. PPARα is poorly expressed in macrophages due to the high levels of IL-1b; but its activation reduces liver inflammation by directly targeting IL-1r antagonist [72]. Oleoylethanolamide, an endocannabinoid-like molecule ameliorated thioacetamide-induced hepatic fibrosis blocking the activation of HSCs inhibiting the expression fibrosis markers, and genes involved in inflammation and extracellular matrix remodeling. These improvements could not be observed in PPARα knockout mice [73].

#### 3.2.2. PPARβ/δ

Contrary to PPARγ role in HSC activation; PPARβ/δ is highly expressed in activated HSCs. In liver injury, PPARβ/δ activation facilitates HSC proliferation by activating p38–JNK–MAPK in CCl4-induced liver fibrosis [74] and augments fibrotic markers expression such as collagen I, α-SMA, TIMPs, and MMPs [75]. PPARβ/δ agonist L165041 and GW501516 increased hepatic expression of fibrosis markers in carbon tetrachloride (CCl4)-injected mice [75,76] and L-165041 increased hepatotoxicity due to HSC activation. Therefore, suppressing PPARβ/δ would be a promising way to avoid fibrosis. PPAR β/δ possesses anti-inflammatory effects in the liver due to direct binding to NF-kB p65 subunit; however, high expression of hepatic proinflammatory factor MCP-1 in CCl4-induced liver disease is associated with PPARβ/δ [77]. PPARβ/δ inhibition reduce liver inflammation through regulation of LPS-mediated TLR4 signaling pathway in cultured hepatocyte cells [78]. PPARβ/δ activation inhibits macrophage activation, showing anti-inflammatory effects [79] and adenoviral over expression of PPARβ/δ in mice decrease JNK signaling and inflammatory markers [80]. Lastly, it has been shown that PPARβ/δ has hepatoprotective effects modulating NF-κB signaling, consequently attenuating CCl_4_ hepatotoxicity [81].

#### 3.2.3. PPARγ

PPARγ is a key factor in HSCs activation and fibrosis pathogenesis. PPARγ can regulate the TGF-β/Smads pathway and binds directly to Smad3 and inhibits TGF-β-induced CTGF and α-SMA expression in smooth muscle cells [82]. Several molecules that upregulate PPARγ can inhibit TGF-β production during fibrosis in different tissues [83,84]. PPARγ is involved in HSC transdifferentiation and fibroblast transformation, PPARγ2 is highly expressed in quiescent HSC, and PPARγ is downregulated during HSC activation [85]. Accordingly, PPARγ restoration prompts the change in activated HSC to quiescent HSC and suppresses activity of AP1 [86]. Most PPARγ agonists can reduce hepatic fibrosis by restraining HSC proliferation and driving activated HSC to apoptosis [87]. In addition, PPARγ can reduce the overexpression of α-SMA, type I collagen, and hydroxyproline and thereby inhibit liver fibrosis [88]. PPARγ ameliorate liver fibrosis and inhibit HSC proliferation regulating many transcription factors, such as CCAAT/enhancer binding protein (C/EBP), LXRα and SREBP-1c, which are depleted when HSCs are activated [89]. PPARγ overexpression could directly reverse liver fibrosis in mice fed with a methionine–choline-deficient (MCD) diet by reducing the expression of α-SMA and tissue inhibitors of metalloproteinases (TIMPs) and increasing HSCs cell apoptosis [90]. Capillarization is a term used to describe when liver sinusoidal endothelial cells (LSECs) lack fenestration and develop an organized basement membrane, which is permissive for HSC activation and is a preamble to fibrosis [91]. PPARγ agonist ameliorates LSECs activation and inflammation [92]; while PPARβ/δ and PPARα agonists induce ICAM-1 expression in non-stimulated ECs playing an important role in liver fibrosis [93].

On the other hand, monocyte-derived macrophages and bone-marrow derived macrophages highly express PPARγ [94]. This nuclear factor induces macrophage M2 polarization, and in consequence an anti-inflammatory liver response. In a CCl_4_-induced liver damage model, null mice for PPARγ in macrophages and HSC showed aggravated liver necroinflammation and fibrosis compared to Pparγ-DHEP mice and control mice demonstrating the important role of alterations in macrophages and HSCs in liver fibrosis [95]. Furthermore, in mice subjected to bile duct ligation rosiglitazone inhibited NF-kB activation and hepatic fibrosis, but these changes disappeared in Pparγ-DHEP mice [96]. Crosstalk was observed between PPARγ and FXR in HSC cells, which was involved in regulating inflammation, contributing to the antifibrotic activity of FXR ligands in rodent liver cirrhosis models [97].

In conclusion, the knowledge of PPARs’ relationship with HSC activation and inflammation will provide a therapeutic strategy for liver fibrosis.

#### 3.2.4. Clinical Trials of PPAR-Related Drugs in Liver Fibrosis

PPARs play an important role in liver fibrosis, by regulating downstream targeted pathways, such as TGF-β, MAPKs, and NF-κB p65. However, no direct clinical trial is registered in in the official database of the U.S. National Library of Medicine (https://clinicaltrials.gov/ct2/home; acceded on 30 July 2021) regarding liver fibrosis, only as part of NASH outcomes.

### 3.3. PPARs in Hepatocellular Carcinoma

HCC is the most common malignant tumor of the liver, and it is the third leading cause of cancer deaths worldwide [98]. However, the survival of patients with late diagnosis is still limited, as many of the therapies are no longer efficient. Therefore, it is important to search for new therapeutic alternatives that, in conjunction with those mentioned above, might help reduce the incidence of HCC. In the following section the role of PPARs in the development of HCC will be described:

#### 3.3.1. PPARα

The role of PPARα has been debated in the past decade. On one hand, several studies postulate that activation of this transcription factor is fundamental for the development of HCC in a wide variety of experimental animal models and in human HCC cells [99,100,101]. However, Xiao YB et al. demonstrated, in a total of 804 samples of human HCC, lower expression of PPARα in the nucleus than in those of normal liver tissue; on the other hand, high expression both in nucleus and in cytoplasm of PPARα correlated with a longer survival time of patients with HCC [102].

The differential localization in the nucleus or cytoplasm may be the answer to the pleiotropic effect of PPARα; however, Thomas et al. postulated that a variant transcript of human PPARα lacks full exon 6 due to alternative splicing, generating a truncated PPARα-tr protein lacking ligand binding domain that cannot binds to PPRE, but is capable of autonomously regulating proliferative and proinflammatory genes [103].

In recent years, increasing obesity and diabetes were related to increase in HCC, yet the molecular mechanism correlating both pathologies has not been elucidated. Senni et al. demonstrated that catabolism of fatty acids through β-oxidation is the main mechanism that allows the use of fatty acids in proliferation through the regulation of β-catenin on PPARα [104].

#### 3.3.2. PPARβ/δ

As mentioned above, the functions of PPARβ/δ overlap with those of PPARα in peripheral tissues, while in the liver its functions are more related to the processes regulated by PPARγ. Liu S et al. showed that the overexpression of PPARβ/δ protects the liver of mice from fatty acid overload; in addition, the inflammatory pathways are also decreased, so the risk of developing HCC is probably reduced [80]. On the other hand, Vacca et al. studied the role of this nuclear factor in the modulation of liver proliferation, confirming the low expression of PPARβ/δ in human HCC and the reduced expression of target genes such as Cpt-1 and TGFβ1. They also verified that the PPARβ/δ agonist GW501516 reduces the proliferative potential of Hepa1-6 hepatoma cells [105].

On the other hand, Kim et al. reported metabolic reprogramming in sorafenib-resistant HCC identifying PPARβ/δ as a key regulator of glutamine metabolism and reductive carboxylation, consequently inhibition of PPARβ/δ activity reversed metabolic reprogramming in HCC cells and sensitized them to sorafenib, suggesting PPARβ/δ as a potential therapeutic target [106].

#### 3.3.3. PPARγ

The expression and activation of PPARγ in HCC has been a controversial issue; however, in recent years, Yu et al. demonstrated that PPARγ expression is significantly reduced in tumor tissue compared to non-tumor liver tissue, particularly in early tumors. Lately, this same research group demonstrated that PPARγ activation suppresses migration and invasion of HCC cells, and can inhibit metastasis in an orthotopic HCC model in vivo [99,107].

Recently, Zuo et al. showed that low levels of expression of peroxisome proliferator-activated receptor gamma coactivator 1α (PGC1α) were associated with a poor prognosis in HCC and revealed the molecular mechanism of PGC1α in the metabolism and progression of HCC. PGC1α suppresses HCC cell metastasis by inhibiting the Warburg effect through regulation of the WNT/β-catenin/PDK1 axis, concluding that the tumor suppressor activity of PGC1α depends on PPARγ [108].

Several co-therapies have been developed aimed at modulating the activity of PPARγ Wang et al., proposed that flavonoid avicularin inhibit cell proliferation, migration and invasion, and changes in cell apoptosis and cell cycle, through positive regulation for PPARγ [109]. For its part. telmisartan can modulate the ERK1/2, TAK1 and NF-κB signaling axis, such as agonist of PPARγ, exerting antitumor effects, and increasing tumor sensitivity to sorafenib [110]. Additionally, Abd-El Baset et al. indicated that β-ionone (βI), a cyclic isoprenoid, can regulate the expression of PPARγ, through ofRXR, proposing β-ionone such as a potential chemotherapeutic agent in combination with sorafenib [111]. Finally, it has been shown that simvastatin can inhibit the HIF-1α/PPARγ/PKM2 axis, suppressing PKM2-mediated glycolysis, decreasing cell proliferation, and increasing the expression of apoptotic markers in HCC cells, sensitizing of them to sorafenib treatment [112].

In conclusion, even though the activation and expression of PPARs in HCC development continues to be controversial, in recent years, complementary therapies have been developed that mainly involve PPARα and PPARγ-activation, sensitizing tumor cells to traditional anticancer treatments used in HCC.

#### 3.3.4. Clinical Trials of PPAR-Related Drugs in HCC

Metronomic chemotherapy is a new modality of drug administration in which there is an administration of conventional chemotherapeutic agents at very low doses target activated endothelial cells in tumor, without the risk of developing adverse effects [113]. A prospective one-arm, multicenter phase II clinical trial evaluated the progression-free survival, safety and tolerability of a metronomic chemotherapy, which included capecitabine, rofecoxib (PPARγ agonist, and PPARβ antagonist) and pioglitazone, a PPARγ agonist in 38 patients with non-curative HCC, and the median progression-free survival was 2.7 months, the median overall survival was 6.7 months [114]. As regards side effects, the most common adverse event was edema grade 3, in 66% of patients [114]. This trial offers interesting results about the efficacy of a biotherapy that includes minimal doses of agonists that modulate the response of PPARs, and its safety, in patients with an advanced stage of HCC. Unfortunately, it is one of the few registered clinical trials where this type of therapy is evaluated in HCC patients.

### 3.4. PPARs in HBV and HCV Infections

Infection with HBV or HCV represents one of the main causes of chronic liver disease in the world. However, in endemic areas, a considerable number of patients are infected with both viruses, mainly as a result of common routes of transmission. Several studies have shown that dual-infected patients have an increased risk of advanced liver disease, fibrosis-cirrhosis, and HCC compared to monoinfected patients. Currently, little is known about the role that PPARs play in the development of the infection [115].

#### 3.4.1. PPARα

PPARα overexpression is characteristic both in the acute and chronic phases of HBV disease; this due to the cccDNA of HBV which has binding sites for global and liver-specific transcription factors such as CCAAT enhancer-binding protein (C/EBP), retinoid X receptor (RXR), and PPARα that bind to enhancer regions I (ENI), Core and Pre-Surface2/Surface promoter proteins [116]. In this manner, after HBV infection there is a PPARα overexpression in hepatocytes characteristic in the G2/M phases of the cellular cycle [117]. Additionally, in the same study performed by Xia et al., they found a negative regulation of TGF-β2 in primary human hepatocytes with HBV infection and TGF-β2 treatment, the levels of PPARα, RXRα, CEBPB mRNA and viral replication decreased significantly [117].

In 2017, Du et al. showed that PPAR agonists such as bezafibrate, fenofibrate and rosiglitazone increase HBV replication, which shows that it is important to analyze viral load in HBV infected patients [118]. Moreover, natural agonists such as resveratrol have a direct effect on Sirtuin-1, promoting PGC1a deacetylation, and this, in turn, supports the transcriptional activity of PPARα, which, according to in vivo and in vitro models, allowed HBV replication [119]. Data of real-time PCR demonstrated that mice knockdown of PPARα or RXRα abolished RSV-induced HBV replication; such a mechanism is clearly dependent on PPARα [119].

HCV infection, through HCV core protein activity, affects the expression and activity of PPARγ in hepatocytes. A decreased expression of this protein is related to the accumulation of lipid droplets in the liver and the eventual development of fatty liver disease [120]. The mechanism is mediated by the HCV core protein, which localizes in the membrane of lipid vesicles and induces hepatic fat accumulation by activating SREBP-1c [120]. One of the mechanisms that explains this effect is through a miRNA. In a study carried out by Shirazaki et al., they infected Huh-7.5 hepatoma cells with a JFH1 strain derived from HCV, finding that this procedure induces the expression of miR-27a [121]. This miRNA targets PPARγ directly, reducing lipid synthesis and increasing lipid secretion; two processes that possibly promote HCV replication and virion efflux [122].

#### 3.4.2. Clinical Trials of PPAR-Related Drugs in Infection HBV/HCV

Despite therapeutic potential of PPARs on HBV/HCV infection, no direct clinical trial is registered in in the official database of the U.S. National Library of Medicine (https://clinicaltrials.gov/ct2/home; acceded on 30 July 2021).

### 3.5. PPARs and Their Role in the Development of ALD

Alcohol is the most socially accepted addictive substance, and its excessive consumption is related to serious health problems [123]. ALD is one of the main causes of death worldwide [124]. This injury is produced by a chronic or binge consumption of ethanol, that is, by ingestion of >40 g or higher of alcohol per day over a prolonged period, or consumption of five standard drinks, 70 g of alcohol in less than 2 h approximately [125]. ALD has a broad spectrum that begins with simple disorders, until more severe forms of liver injury develop. Accumulation of fat in the liver, induced by alcohol consumption (AFL), or steatosis, is the earliest response, and 80–90% of chronic alcohol drinkers develop this damage process; this injury can be reversible through exercise, a low fat-calorie diet consumption, or alcohol withdrawal [125,126]. If noxious stimuli continue, liver damage progresses to an inflammatory lesion, known as alcoholic hepatitis, where only 20–40% of chronic consumers develop it, and can slowly progress to steatohepatitis, fibrosis, cirrhosis, and eventually to HCC [127]. Several risk factors have been identified such as gender, where women are more likely to develop ALD, since there are lower levels of gastric alcohol dehydrogenase, in addition to the presence of a higher proportion of body fat [128,129]. Genetic variants are other risk factors that allow ALD progression, studies demonstrate that variations in patatin-like phospholipase domain-containing protein 3 (PNPLA3), trans-membrane 6 superfamily member 2 (TM6SF2) and membrane-bound O-acyltransferase domain-containing protein 7 (MBOAT7) are important genetic determinants of risk and severity of ALD. Although their mechanisms and responses are not entirely clear, mutations in these genetic variants seem to be related to lipid metabolism [125,130,131]. Finally, a co-infection with hepatitis virus B or C can accelerate progression of ALD to liver fibrosis, cirrhosis, or HCC [132].

#### 3.5.1. PPARα

The first alteration that occurs after excessive alcohol is an increase in the proportion of reduced nicotinamide adenine dinucleotide (NADH) and oxidized nicotinamide adenine dinucleotide (NAD+) in hepatocytes [133]. Ingested ethanol is metabolized through the activity of the cytosolic alcohol dehydrogenase enzyme in acetaldehyde, and subsequently in acetate through the participation of the mitochondrial aldehyde dehydrogenase enzyme. Both enzymes use NAD+ as a co-factor, and in response NADH is produced in both steps [134] An increase in NADH levels results in a disruption of mitochondrial β-oxidation of fatty acids, an alteration in energy supply and an increase in fatty acid formation, allowing AFL development [133,134].

Currently, several key molecular mechanisms have been identified as triggers for the AFL development after excessive alcohol intake; one of them is regulated by an increase in the expression of SREBP-1c, and on the other hand, by the decrease in the expression of PPARα. The latter allows ALF generation via fatty acid synthesis induction, and fatty acid-β-oxidation inhibition [135].

Acetaldehyde can inhibit PPARα activity through its covalent binding to the transcription factor, consequently, the binding of PPARα to a specific DNA sequence is also inhibited [136]. On the other hand, alcohol can indirectly block PPARα activation by oxidative response generated by upregulation of cytochrome P450 2E1 activity [137].

In a study carried out by Nakajima et al., it was observed that PPARα knockout mice administered with a liquid diet containing 4% ethanol, exhibited hepatomegaly, inflammation, apoptosis, and fibrosis [138]. RXR function is also affected by the consumption of ethanol. Feeding mice with ethanol showed a decrease in the levels of RXRα protein, which in turn did not allow binding of PPARα/RXRα to DNA, decreasing mRNA for several genes regulated by PPARα, and therefore, the development of steatosis was favored [139].

Information demonstrating that PPARα agonist administration improves hepatic injury induced by alcohol consumption has been generated. In experimental studies with C57BL/6 mice fed with ethanol and treated with WY14643, a PPARα agonist, fat accumulation in the liver was prevented [139,140]. Recently, the hepatoprotective effect of Danshen, a traditional Chinese medicine compound, was evaluated in an experimental model of alcoholic liver damage using male C57BL/6 mice, and in an in vitro model. Danshen was effective in preventing ALD through activation of PPARα and reducing 4-hydroxynonenal levels [141]. Other natural compound that has been shown to be effective in preventing alcoholic liver damage is *Solanum muricatum Ait* (pepino fruit), a common plant cultivated in Taiwan. In an animal model of alcoholic liver damage this compound was effective in improving lipid accumulation induced by ethanol, and the molecular evaluation showed that this response is mediated through induction of hepatic levels of p-AMPK and PPARα; also, this compound reduced SREBP-1c expression, an important hepatic lipogenic enzyme [142].

#### 3.5.2. Clinical Information about PPARs Activity in ALD

Fibrates are PPARα agonists used to treat problems such as dyslipidemia and hypercholesterolemia; however, there is various evidence that demonstrate its effectiveness in reducing alcohol consumption in mice and rats [143,144]. On the other hand, Muñoz et al., in 2020, demonstrated that treatment with Fenofibrate (100 mg/kg) was effective in producing an increase in the expression and activity of the protein alcohol dehydrogenase 1, showing an additional pharmacological mechanism of action to counteract liver damage due to alcohol consumption [145].

Other agonists of these nuclear receptors such as pioglitazone, rosiglitazone, and ciglitazone also have beneficial effects in reducing alcohol consumption [146]; nevertheless, none of the available studies have focused on elucidating the mechanisms by which these agonists can improve liver functionality after chronic damage due to alcohol consumption in humans. Regarding clinical trials, at the present there are no trials registered in in the official database of the U.S. National Library of Medicine (https://clinicaltrials.gov/ct2/home; acceded on 30 July 2021) related with PPARs agonists and ALD patients. This represents an opportunity area to explore the efficacy and safety of PPARα agonist drugs in patients with ALD.

In conclusion, ALD is a pathology responsible for the morbidity and mortality of millions of people around the world. The first harmful response that occurs is steatosis, which occurs in more than 80% of people who consume alcohol in a chronic way. In this phase, the role played by PPARα has allowed the understanding of mechanisms of damage that occurs after alcoholic intake. Agonists of PPARα have demonstrated efficacy at the pre-clinical level to prevent development of alcoholic liver disease in its most advanced stages; however, it is necessary to continue studying their effects and safety in clinical studies.

## 4. Conclusions and Perspectives

**Figure 3:**
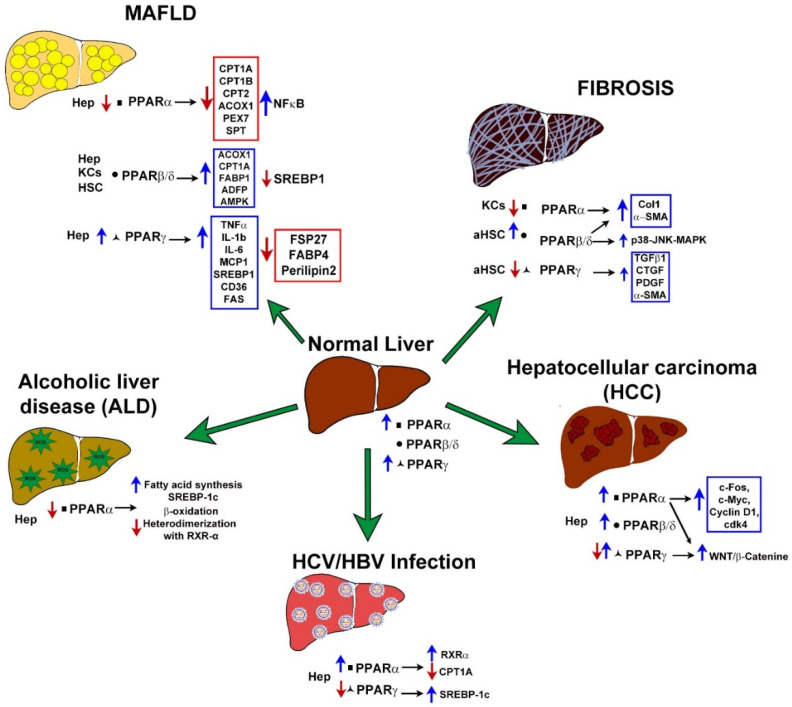
Main molecular targets regulated by PPARs in liver diseases addressed in this review: metabolism-associated fatty liver disease (MAFLD), alcoholic liver disease (ALD), fibrosis, HBV viral hepatitis or HCV infection and hepatocellular carcinoma (HCC). Blue arrows indicate over-activation, while red arrows indicate downregulation of triggered responses. Hep, hepatocytes; KCs, Kupffer Cells; HSCs, Hepatic Stellate Cells.

Liver disease continues to be a challenge to health systems worldwide. In previous years, the search for new therapeutic strategies was focused on the study of fibrogenic processes, and the role of HSC. However, in recent years the efforts of liver disease professionals have focused on the study of early stages of the disease, where accumulation of lipids and metabolic alterations are key processes for the development of these diseases. Figure 3 and Table 2 summarize the role of PPARs as metabolic sensors in different liver diseases.

Since the first description of PPARs [148], our knowledge about these nuclear factors has been increasing. At first, PPARs were only considered as regulators of lipid metabolism; however, currently they are considered the main hepatic metabolic mediators, having an important role in various processes such as: cell survival, regulation of ubiquitination, adipocyte differentiation, regulation of thermogenesis and gluconeogenesis mediators. Taking the above into account, the design and study of new pharmacological therapies for the treatment of liver diseases should be aimed at modulating the activity of these transcription factors.

Finally, the use of liver-specific PPAR-null mice has opened the possibility of studying other important mechanisms in which PPARs are involved [149], mainly as mediators of epigenetic regulation mechanisms through their interaction with enzymes such as Sirtuin-1 [32,150], the regulation of PPAR promoters, through DNA methyltransferases (DNMTs) [151], and the regulation of their expression through a variety of microRNAs [152].

## Figures and Tables

**Table 1:** Main characteristics of PPAR subtypes.

PPAR Subtype	PPARα	PPAR β/δ	PPARγ
Gene location	Human chromosome 22q12.2–13.1	Human chromosome 6p21.1–21.2	Human chromosome 3p25
Isoforms	None	None	PPARγ1, PPARγ2, PPARγ3
Tissue distribution	Liver, heart, skeletal muscle (tissues with high fatty acid oxidation rates); brown adipose tissue, kidney, adrenal gland.	Liver, kidney, skeletal and cardiac muscle, adipose tissue, brain, colon, vasculature, esophagus, gut. Ubiquitous.	Mainly in adipose tissue (white and brown). Other tissues such as liver, gut, kidney, retina, immunologic system, muscles, spleen, urinary bladder, heart, lung, brain, vasculature.
Endogenous Ligands	Unsaturated and saturated fatty acids and their derivatives (8-S-hydroxyeicosatetraenoic acid, arachidonic acid lipoxygenase metabolite LTB4, arachidonate monooxygenase metabolite epoxyeicosatrienoic acids), leukotriene derivatives, VLDL hydrolysis products.	Unsaturated fatty acids, arachidonic acid cyclooxygenase metabolite prostacyclin, the linoleic acid 15-lipoxygenase-1 product 13-S-hydroxyoctadecadienoic acid, carbaprostacyclin, components of VLDL.	Polyunsaturated fatty acids, prostanoids (15-deoxy-Δ12, 14-prostaglandin J2 (15-dPGJ2)), 13-hydroxyeicosatetraenoic acid, components of oxidized LDL, eicosanoids, oxidized alkyl phospholipids.
Functions	Major regulator of the mitochondrial and peroxisomal β-oxidation (fatty acid metabolism), lowers lipid levels, anti-inflammatory activities.	Increase lipid catabolism, improves the plasma HDL-cholesterol levels and insulin resistance, induce cell proliferation and differentiation, anti-inflammatory activities.	Regulate adipocyte differentiation, lipid storage, and glucose metabolism (improves insulin sensitivity), main regulator of metabolic genes, increase fatty acid oxidation, HDL and uncoupling protein, decrease triglycerides, improves vascular integrity, energy balance, anti-inflammatory activities.
Target genes	CYP8B1, FATP, FAT/CD36, liver cytosolic FABP, LPL. Lipid/hormone transport genes. (LEPR, SLC27A2, SLC27A4). Acyl-CoA metabolism. (ACOT12, ACSL3, ACSL3, ACSL5, ACSL1, ACSM3, FABP1, FABP3). β-oxidation. (ACAA2, ACADM, ACADS, ACADVL, CPT1A, CPT2, ETFDH, HADHA, HADHB, SLC25A20, SLC22A5, TXNIP). Ketogenesis/ketolysis genes. (FGF21, HMGCS2), Peroxisomal β-oxidation (ABCD2, ABCD3, ACAA1A, ACOX1, ECH1, HSD17B4). Lipogenesis. (ACACB, AGPAT2, ELOVL5, ELOVL6 FADS1, GPAM, MLYCD, MOD1). Lipases and lipid droplet proteins. (ADFP, CIDEC, PNPLA2, S3-12). Lipoprotein metabolism. (ANGPTL4, APOA1, APOA2, APOA5, APOCIII, LIPC, PCTP, VLDLR). Cholesterol and bile metabolism. (ABCA1, ABCB4, CYP7A1, FXR, LXR)	Genes related with lipid uptake, represses genes that participated in lipid metabolism and efflux. LPL, PGAR, IDK, PDK-1, Ubiquitin C, CPT1, AOX, LCAD, UCP1, UCP3, PGC1-alpha. Tumor angiogenesis (Pdgfrβ, Pdgfb, c-kit)	AP2, CAP, IRS2, GLUT4, GLUT2, adiponectin, ACS, PCK2, LPL, FAT/CD36, FABP, GYK fatty acid transport, acyl-CoA synthetase, glucokinase, leptin, perilipin, GK PEPCK, UCP 1, UCP-2, UCP-3, LXR-alpha, TNF-alpha, IL-6.
References	[8,10,11,12,13,14,15]	[8,10,11,12,13,14,16,17]	[8,10,11,12,13,14,17,18,19,20]

HDL, High density lipoprotein; LDL, low density lipoprotein; VLDL, Very low-density lipoprotein.

**Table 2:** Role of PPARs in liver diseases.

Liver Disease	Expression		Function	Mutation	Reference
MAFLD	Hepatocytes Kupffer Cells Hepatic Stellate Cells	↓ PPARα ↑ PARβ/δ ↑ PPARγ ↑ PARβ/δ ↑ PPARγ	PPARα: Induces lipogenesis PPARβ/δ: Augments liver fat content and decreases insulin sensitivity PPARγ: Promotes steatosis	PPARA: CM003689 association with elevated plasma lipid concentration in diabetes CM025499 CM025500 associated with diabetes PPARG: CM981614, CM981615, CM1617313 associated with Obesity CM066185, CM066187, CM066186, CM066188, CD066392, CX022192 Associated with IR CR032439 association with increased height/lipid metabolism CR057908 association with increased body weight	[25,47,62] The Human Gene Mutation Database, consulted July 2021
Fibrosis	Kupffer Cells Hepatic Stellate Cells (activated)	↓ PPARα ↑ PPARβ/δ ↓ PPARγ	PPARα: Increases oxidative stress and inflammation PPARβ/δ: Facilitates HSC activation PPARγ: key factor in HSCs activation and regulation of inflammation	No mutations associated with liver fibrosis	[73,74,90]
Hepatocellular carcinoma	Hepatocytes	↑ PPARα ↑ PARβ/δ ↓↑PPARγ	PPARα: Regulates expression of B-catenin, c-Fos, c-Myc, Cyclin D1. PPARγ: Modulates activity of p53, ERK1/2, TAK1 and NF-κB	PPARG: R280C, C285Y, Q286P, F287Y, R288C, R288H S289C mutations are potential loss offunction mutations in various aspects including ligand binding for PPARγ activation	[104,105,106,147]
HBV and HCV infections	Hepatocytes	↑ PPARα ↓ PPARγ	PPARα: Increase fatty acid Synthesis PPARγ: Decrease β-oxidation	No mutations associated with HBV, or HCV infection	[116,117,118,119]
Alcoholic liver disease	Hepatocytes	↑ PPARα	PPARα: Increase fatty acid Synthesis Decrease β-oxidation	No mutations associated with alcoholic liver disease	[134,135,136,137]

## Data Availability

The data used to support this study are included within article as references.
